# Supplementary material for: Shaded tangles for the design and verification of quantum circuits
Source: Proc Math Phys Eng Sci. 2019 Apr 3;475(2224):20180338. doi: 10.1098/rspa.2018.0338 (PMC6501654; doi:10.1098/rspa.2018.0338)
Supplement: Appendices [file rspa20180338supp1.pdf]

A Reidemeister III Hadamard matrices

The additional RIII condition (2.3) induces substantial constraints on a self-transpose Hadamard matrix. Here, we show that these equations have solutions in all finite dimensions. We consider two different families of solutions: *Potts-Hadamard matrices*, and *metaplectic invariants*. Almost everything in this section follows directly from results of Jones [41] on building link invariants from statistical mechanical models.

Potts-Hadamard matrices. A *Potts-Hadamard matrix* is a self-transpose Hadamard matrix of the following form, that satisfies (2.3):

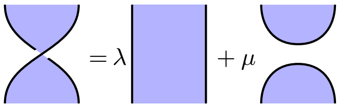

(A 1)

In tensor notation, this means that $H_{a,b} = \lambda\,\delta_{a,b} + \mu$. We can classify Potts-Hadamard matrices exactly.

Theorem A.1. *Every Potts-Hadamard matrix has* $\mu = \frac{1}{\sqrt{d}}\overline{\lambda}$ *with*

$$\lambda \in U(1) \quad \textit{and} \quad \lambda^2 + \overline{\lambda}^2 = -\sqrt{d} \tag{A 2}$$

where d is the dimension of the Hadamard matrix. This has the following solutions:

- $d = 2$ *and* $\lambda \in \{e^{\frac{3\pi i}{8}}, e^{-\frac{3\pi i}{8}}, e^{-\frac{5\pi i}{8}}, e^{\frac{5\pi i}{8}}\}$;
- $d = 3$ *and* $\lambda \in \{e^{\frac{5\pi i}{12}}, e^{-\frac{5\pi i}{12}}, e^{-\frac{7\pi i}{12}}, e^{\frac{7\pi i}{12}}\}$;
- $d = 4$ *and* $\lambda \in \{i, -i\}$.

The $d = 2$ Potts-Hadamard matrices have the following form:

$$\frac{e^{-\frac{\pi i}{8}}}{\sqrt{2}}\begin{pmatrix} 1 & i \\ i & 1 \end{pmatrix} \qquad \frac{e^{\frac{\pi i}{8}}}{\sqrt{2}}\begin{pmatrix} 1 & -i \\ -i & 1 \end{pmatrix} \qquad \frac{e^{\frac{7\pi i}{8}}}{\sqrt{2}}\begin{pmatrix} 1 & i \\ i & 1 \end{pmatrix} \qquad \frac{e^{-\frac{7\pi i}{8}}}{\sqrt{2}}\begin{pmatrix} 1 & -i \\ -i & 1 \end{pmatrix}$$

In fact, it can be shown by direct calculation that these are the only two dimensional self-transpose Hadamard matrices fulfilling (2.3).

Equation (A 1) (together with (A 2)) is a rescaled version of the defining relation of Kauffman's bracket polynomial [46]; evaluating one of these matrices on a closed link diagram therefore yields (after suitable renormalization) the Jones polynomial of the link at certain roots of unity.

Metaplectic invariants. Following Jones and others [29, 37, 41], given $d \in \mathbb{N}$ with $d > 0$, we make the following definitions:

$$\xi := -e^{\frac{\pi i}{d}} \qquad\qquad \omega := \tfrac{1}{\sqrt{d}} \textstyle\sum_{k=0}^{d-1} \xi^{k^2} \tag{A 3}$$

Let λ be a square root of ω, and for $0 \le a, b \le d-1$, define $H_{a,b}$ as follows:

$$H_{a,b} = \frac{\overline{\lambda}}{\sqrt{d}}\,\xi^{(a-b)^2} \tag{A 4}$$

Then we have the following.

Theorem A.2. *The coefficients $H_{a,b}$ define a self-transpose Hadamard satisfying* (2.3).

This establishes that solutions to our graphical equations can be found in all finite dimensions.

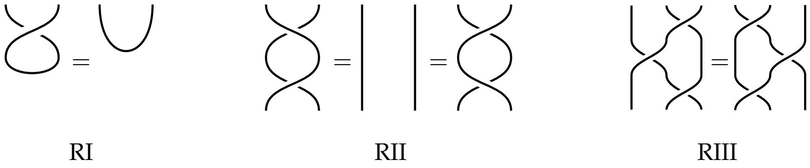

Figure 25: The unshaded Reidemeister moves up to rotations and reflections.

B Omitted proofs

The following basic theorem shows that our extended calculus yields a shaded variant of the tangle calculus. The argument is straightforward and certainly not original; for similar work, see Kauffman [49] and Cordova et al [20].

Theorem 2.1. *Two shaded tangle diagrams with the same upper and lower boundaries are equal under the axioms of the extended calculus (up to an overall scalar factor) just when their underlying tangles, obtained by ignoring the shading, are isotopic as classical tangles.*

Proof. It is well known that two tangle diagrams are isotopic just when they can be transformed into each other using local Reidemeister moves [49]. All Reidemeister moves can be obtained from arbitrary rotations and reflections of the moves depicted in Figure 25. Since our tangles are shaded, they transform under *shaded Reidemeister moves*, ordinary Reideimeister moves with a choice of checkerboard shading. Thus, up to rotations and reflections, there are 2 shaded versions of RI, 4 shaded versions of RII and 2 shaded versions of RIII. To prove Theorem 2.1, we therefore have to show that (up to scalar factors) all these shaded Reidemeister moves are implied by the basic axioms of the extended calculus presented in Figure 6. Using the shaded RII equations, it can be shown that the two shaded RIII equations are equivalent. Using shaded RII and RIII, it can be shown that the two shaded RI equations are equivalent. Therefore, two shaded tangles are isotopic if and only if they can be transformed into each other using all four shaded RII equations and one shaded RI and RIII equation, respectively. □

Theorem 2.2. *In* **2Hilb***, a shaded crossing yields a solution of the basic calculus just when it is equal to a self-transpose Hadamard matrix.*

Proof. Solutions to the shaded Reimeister II equations in **2Hilb** Figure 6(b) and (c) were classified in terms of Hadamard matrices in [68, Proposition 7]. The additional equation Figure 6(d) implies that the corresponding Hadamard matrix is self-transpose. An equivalent classification using slightly different terminology can be found in [41]. □

Theorem 2.3. *In* **2Hilb***, a self-transpose Hadamard matrix satisfies the extended calculus just when:*

$$\sum_{r=0}^{|S|-1} \overline{H}_{ar} H_{br} H_{cr} = \sqrt{|S|}\, \overline{H}_{ab} \overline{H}_{ac} H_{bc} \tag{2.3}$$

Proof. Translating the Reimeister III equation Figure 6(f) into the corresponding family of tensor diagrams (as described in Figure 4) yields the following:

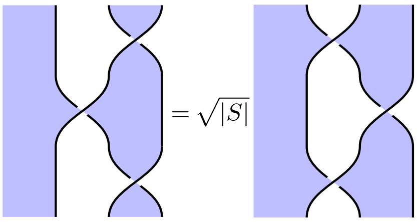

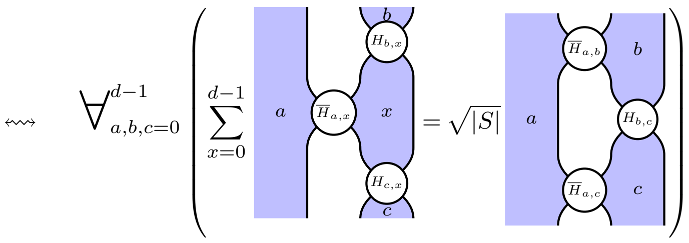

Here a, b, and c label the left, top right, and bottom right shaded region, respectively. The central shaded region is labelled by x and summed over. Note that the Hadamard matrix H is self-transpose. Thus, this results in equation (2.3). Similarly, the Reidemeister I equation Figure 6(e) translates into the following equation which is a direct algebraic consequence of (2.3) for $a = b$ (with $\lambda = \sqrt{|S|}\,\overline{H}_{a,a}$): $\sum_{r=0}^{|S|-1} H_{c,r} = \lambda$. An equivalent classification using slightly different terminology can be found in [41]. □

Theorem A.1. *Every Potts-Hadamard matrix has* $\mu = \frac{1}{\sqrt{d}}\overline{\lambda}$ *with*

$$\lambda \in U(1) \quad \textit{and} \quad \lambda^2 + \overline{\lambda}^2 = -\sqrt{d} \tag{A 2}$$

where d is the dimension of the Hadamard matrix. This has the following solutions:

- $d = 2$ *and* $\lambda \in \{e^{\frac{3\pi i}{8}}, e^{-\frac{3\pi i}{8}}, e^{-\frac{5\pi i}{8}}, e^{\frac{5\pi i}{8}}\}$;
- $d = 3$ *and* $\lambda \in \{e^{\frac{5\pi i}{12}}, e^{-\frac{5\pi i}{12}}, e^{-\frac{7\pi i}{12}}, e^{\frac{7\pi i}{12}}\}$;
- $d = 4$ *and* $\lambda \in \{i, -i\}$.

Proof. For a shaded crossing of the form (A 1) the two Reidemeister II equations look as follows:

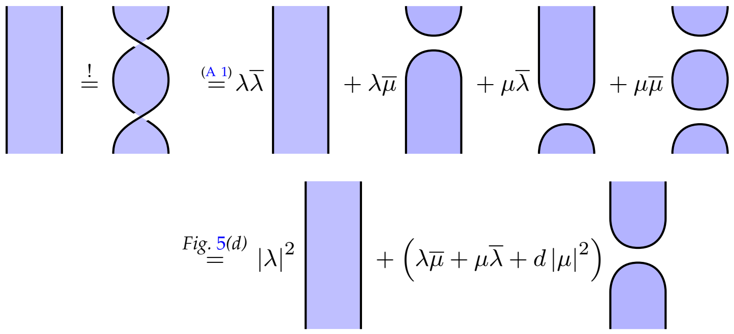

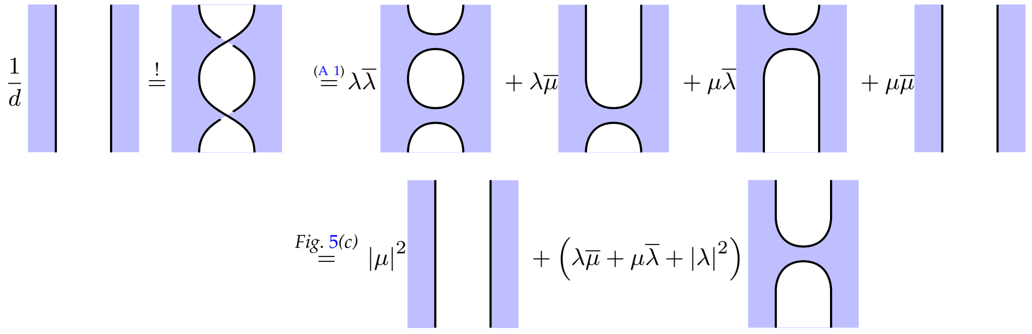

In other words, $|\lambda| = 1,\ |\mu| = \frac{1}{\sqrt{d}}$, and $\lambda\overline{\mu} + \mu\overline{\lambda} = -1$. Reidemeister III yields the following:

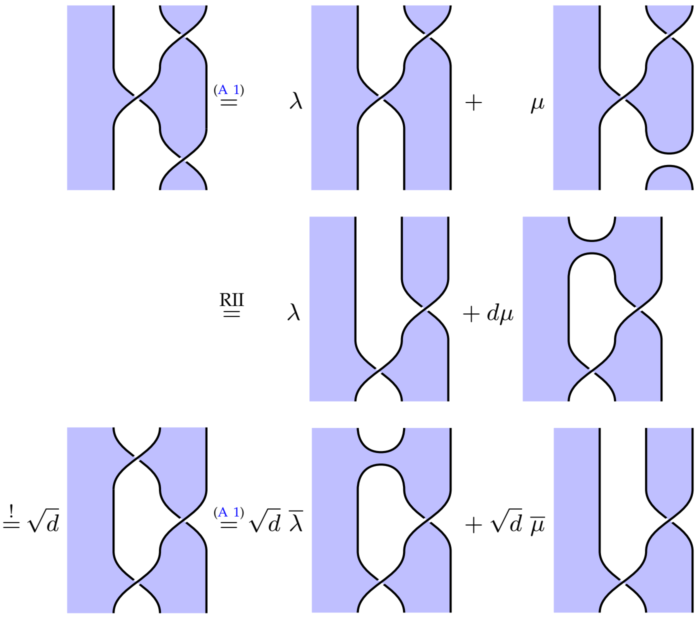

In short, $\mu = \frac{\overline{\lambda}}{\sqrt{d}}$. Together with the constraints from RII this proves the theorem. □

Theorem A.2. *The coefficients $H_{a,b}$ define a self-transpose Hadamard satisfying* (2.3)*.*

Proof. Note that ω (defined in (A 3)) and its square root λ have modulus one. A proof of this fact using the discrete Fourier transform can be found in [37, Proposition 2.15]. Therefore, $|H_{a,b}| = \frac{1}{\sqrt{d}}$. The matrix H is unitary, since

$$\sum_{c=0}^{d-1} H_{a,c}\overline{H}_{b,c} \overset{(A\,4)}{=} \frac{1}{d}\sum_{c=0}^{d-1}\xi^{(a-c)^2-(b-c)^2} = \frac{1}{d}\xi^{a^2-b^2}\sum_{c=0}^{d-1}\xi^{2bc-2ac} \overset{(A\,3)}{=} \frac{1}{d}\xi^{a^2-b^2}\sum_{c=0}^{d-1} e^{\frac{2\pi i}{d}(b-a)c} = \delta_{a,b}.$$

It satisfies (2.3), since

$$\begin{aligned}\sum_{r=0}^{d-1} \overline{H}_{a,r}H_{b,r}H_{c,r} &\overset{(A\,4)}{=} \frac{\overline{\lambda}}{d^{\frac{3}{2}}}\sum_{r=0}^{d-1}\xi^{-(a-r)^2+(b-r)^2+(c-r)^2} = \frac{\overline{\lambda}}{d^{\frac{3}{2}}}\xi^{b^2+c^2-a^2}\sum_{r=0}^{d-1}\xi^{r^2+2r(a-b-c)} \\ &= \frac{\overline{\lambda}}{d^{\frac{3}{2}}}\xi^{b^2+c^2-a^2-(a-b-c)^2}\sum_{r=0}^{d-1}\xi^{(r+(a-b-c))^2} = \frac{\overline{\lambda}}{d^{\frac{3}{2}}}\xi^{b^2+c^2-a^2-(a-b-c)^2}\sum_{r=0}^{d-1}\xi^{r^2} \qquad \text{(A 1)} \\ &\overset{(A\,3)}{=} \frac{\lambda}{d}\xi^{-2a^2+2ab+2ac-2bc}.\end{aligned}$$

The second equality in (A 1) holds since $\xi^{d^2} = 1$. On the other hand,

$$\sqrt{d}\,\overline{H}_{a,b}\overline{H}_{a,c}H_{b,c} = \frac{\lambda}{d}\xi^{-(a-b)^2-(a-c)^2+(b-c)^2} = \frac{\lambda}{d}\xi^{-2a^2+2ab+2ac-2bc}.$$

Thus, H is a self-transpose Hadamard matrix fulfilling (2.3).

□
